# Punctuation, Prosody, and Discourse: Afterthought Vs. Right Dislocation

**DOI:** 10.3389/fpsyg.2015.01803

**Published:** 2015-12-01

**Authors:** Janina Kalbertodt, Beatrice Primus, Petra B. Schumacher

**Affiliations:** ^1^IfL Phonetik, University of CologneCologne, Germany; ^2^Department of German Language and Literature I, University of CologneCologne, Germany

**Keywords:** punctuation, discourse structure, prosody, syntax, right dislocation, afterthought

## Abstract

In a reading production experiment we investigate the impact of punctuation and discourse structure on the prosodic differentiation of right dislocation (RD) and afterthought (AT). Both discourse structure and punctuation are likely to affect the prosodic marking of these right-peripheral constructions, as certain prosodic markings are appropriate only in certain discourse structures, and punctuation is said to correlate with prosodic phrasing. With RD and AT clearly differing in discourse function (comment-topic structuring vs. disambiguation) and punctuation (comma vs. full stop), critical items in this study were manipulated with regard to the (mis-)match of these parameters. Since RD and AT are said to prosodically differ in pitch range, phrasing, and accentuation patterns, we measured the reduction of pitch range, boundary strength and prominence level. Results show an effect of both punctuation and discourse context (mediated by syntax) on phrasing and accentuation. Interestingly, for pitch range reduction no difference between RDs and ATs could be observed. Our results corroborate a language architecture model in which punctuation, prosody, syntax, and discourse-semantics are independent but interacting domains with correspondence constraints between them. Our findings suggest there are tight correspondence constraints between (i) punctuation (full stop and comma in particular) and syntax, (ii) prosody and syntax as well as (iii) prosody and discourse-semantics.

## Introduction

Right dislocation (RD) and afterthought (AT) are two constructions at the right sentence periphery which have often been confused in previous research. Examples are given in (1–2):

1. Ich habe gehört, du magst Peter gern. – Das tue ich. Ich habe ihn neulich getroffen, den Peter.
  “I heard you like Peter a lot. – I do. I've met him recently, Peter.” (RD)
2. a. Kennst du Stefan und Thomas? – Ich habe ihn neulich getroffen. Den Thomas, meine ich.
  “Do you know Stefan and Thomas? – I've met him recently. Thomas, I mean.” (AT)
  b. Kennst du die Frau und das Model? – Ich habe sie eben getroffen. Das Model, meine ich.
  “Do you know the woman (FEM) and the model (NEU)? – I've just met her (FEM). The model (NEU), I mean. (AT)

Only in the last few decades it has become clear that RD and AT are distinct in several respects and need to be kept apart. In the following we will show that RD and AT can be distinguished in terms of morpho-syntax, discourse structure, prosody, and punctuation.

In terms of morpho-syntax, the two dislocations can be distinguished as follows (cf. Lambrecht, 2001; Dewald, 2014). In RD [see *Peter* in example (1)], the dislocated phrase has a coreferential pronoun in the matrix clause that is subject to obligatory gender, number and case agreement. Accordingly, RDs may be analyzed as syntactically connected to the matrix clause. Their special status can be captured by assuming that this connection is not a proper head-dependent relation. ATs in turn [like *Thomas* in (2a)] do not need obligatory agreement in terms of gender with a coreferential pronoun [see *sie* (FEM) – *das Model* (NEU) in (2b)]. Obligatory vs. optional gender agreement also distinguishes restrictive relative clauses from non-restrictive, parenthetical ones (cf. the experimental study in Kirchhoff and Primus, 2014). Thus, ATs, like non-restrictive relative clauses, may be analyzed as being syntactically disconnected from the matrix clause. In addition, ATs but not RDs may be separated from the matrix clause, for instance by parentheticals. ATs may also be accompanied by an illocutionary marker [e.g., *I mean* in (2)], indicating that ATs have their own illoctionary force. Since we are interested in the syntax-prosody interface, we propose a rudimentary syntactic analysis for RDs and ATs that uses the term *root clause* as in the pertinent intonational research (e.g., Truckenbrodt, 2007). A root clause (or comma phrase in Selkirk, 2005) is a syntactic phrase that is not dominated by a higher syntactic node and that has its own illocutionary force. Since a root clause is not necessarily a complete sentence, we will use the term *root phrase* in the following. What counts for our purposes is that ATs but not RDs form their own root phrase, as schematically illustrated in (3).

1. a. RootP[matrix clause] RootP[XP] (AT)
  b. RootP[[matrix clause] [XP]] (RD)

At the discourse level, RD and AT serve different discourse functions (see e.g., Averintseva-Klisch, 2009 for German, Lambrecht, 1981 for French, Fretheim, 1995 for Norwegian). RD, on the one hand, creates a comment-topic relation, which is used to emphasize the importance of the comment, and its use is only appropriate in contexts where the RD-constituent has already been established as topic [see example (4)^1^ where the Dutch team represents the topic—here defined as what is being talked about]. Thus, RDs are best analyzed as contextually given topics. The AT-constituent, on the other hand, has usually been mentioned before [see example (5)] but can be used even if it has not yet been established as discourse topic. ATs are used to resolve an ambiguous reference made earlier in the discourse. Thus, ATs select a referent from a set of contrasting alternatives.

1. und **holland** isch ja die mannschaft sagn wa mal international **die**.. in **ihrer** sagn wa mal in **ihrer** spielweise sagn wa mal am.. nachhaltigsten sind. also **die** schon seit.. ewigkeiten immer einfach immer das gleiche system spielen. das können **sie** sehr sehr gut. isch manchmal vielleicht auch n nachteil aber.. **sie** können auch schon klasse fußballspielen **die holländer**.
  “and **the Netherlands** are the team, let's say, internationally, **that**…in **their**, let's say, in **their** way of playing, let's say,.. is most consistent. well, **they** have been employing the same strategy for.. ages. always, just always playing the same system. that is what **they** are really, really good at. this sometimes might be a disadvantage but…**they** can play really nice soccer, **these Dutchmen**.”
2. **er** sitzt auf der elf seh ich grade. ja ja **er** sitzt auf der elf…da is äh…die zehn zu sehen aber **er** sitzt auf der elf. *sturm* mit einem wahnsinns finish jetzt. kommt *er* da noch mal ran? noch vierundreißig sekunden.…also das wär natürlich der oberknaller.. wenn ***er*** hier zu lange gewartet hätte… **der thomas**.
  **“he** is sitting on [box] eleven, I only just recognized. yes, yes, **he** is sitting on [box] eleven…you can see.. um.. number ten but **he**'s sitting on [box] eleven. [*Felix*] *Sturm* with an incredible finish, now. can *he* make it after all? only 34 seconds left.…. well, this would be astonishing.. if ***he*** had waited too long… **(the) Thomas**.”

**Figure 1:**
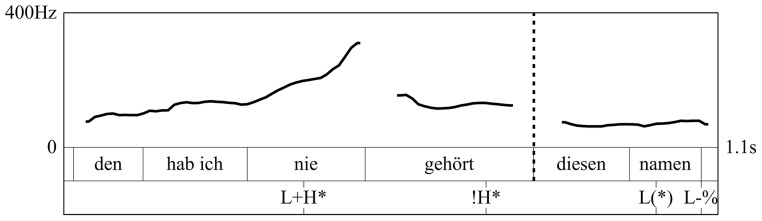
**Tonal contour of a German RD (translation: “I've never heard it, this name”)**. The graph shows the f0-contour, the word level annotation and the GToBI annotation, where the ‘^*^’ indicates which tonal event is associated with the prominent syllable. Stars in brackets [‘(^*^)’] mark postnuclear prominences. The dashed line marks the onset of the dislocated phrase (“this name”)^3^.

**Figure 2:**
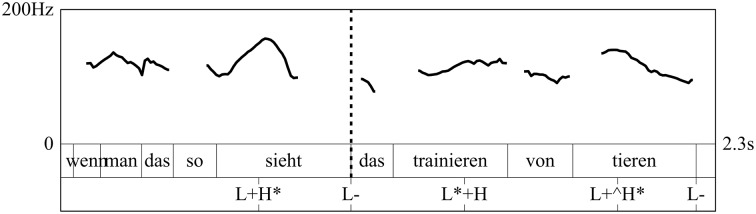
**Tonal contour of a German AT (translation: “When you observe it, the training of animals”)**. The graph shows the f0-contour, the word level annotation and the GToBI annotation, where the ‘^*^’ indicates which tonal event is associated with the prominent syllable. Stars in brackets [‘(^*^)’] mark postnuclear prominences. The dashed line marks the onset of the dislocated phrase (“the training of animals”) at which also a prosodic phrase boundary might be produced^4^.

In terms of prosody, RDs are typically produced either without a phrase boundary or with a weaker intermediate phrase (ip) boundary preceding the dislocated constituent. This way, the dislocated phrase is always produced within the same intonational phrase as the matrix clause (cf. Dewald, 2014), as opposed to AT, which is always segregated from the matrix clause by a stronger intonation phrase (IP) boundary (cf. Dewald, 2014) and thus produced in a separate intonation phrase^2^. Moreover, following Lambrecht (2001), RDs are assumed to be prosodically marked by a reduction of pitch range (Figure 1), which results in a flat intonation contour, while ATs are assumed to not undergo this pitch range reduction but rather use the full pitch inventory (Figure 2).

The intonational phrasing difference between RD and AT can be explained at the prosody-syntax interface by universal alignment- or matching-constraints (cf. Selkirk, 2005, 2011) requiring the right/left edge of every root phrase to coincide with the right/left edge of an IP (Align-RootP). Recall that ATs, but not RDs form an independent root phrase. In addition, a general violable Align-Clause constraint is operative in German (cf. Truckenbrodt, 2005; Selkirk, 2011). It requires that the right edge of every (main or embedded) clause coincides with the right edge of an IP. This constraint may lead to an IP-boundary before an RD. However, its effects are overridden by discourse structure. Since both syntax and discourse structure determine prosodic representations, we follow recent approaches in assuming that we need syntactic representations enriched with discourse structural information in order to explain prosody (e.g., Selkirk, 2011). Discourse structure affects prosody, e.g., by information status in terms of givenness, contrast, topic-comment, etc. Crucially, RDs are considered contextually given topics (cf. e.g., Averintseva-Klisch, 2009). Givenness typically leads to deaccentuation in West Germanic languages (cf. Grice and Baumann, 2007) and thus RD-elements will be deaccented in German. Since deaccented phrases cannot form an own IP (cf. Crystal, 1969), the RD will be prosodically integrated into the IP of the matrix clause. By contrast, the AT-element will never be deaccented, because ATs select a referent from contrasting alternatives, as mentioned above. Contrastively used elements are accented and form their own IP (e.g., Selkirk, 2005). This implies a correspondence between prosodic boundary types and accent strength. In sum, syntax and discourse structure predict a prosodic phrasing as illustrated in (6). This prediction is in line with previous empirical studies on RDs and ATs.

1. a. _IP_[matrix XP] (RD)
  b. _IP_[matrix] _IP_[XP] (AT)

A previous production study (Kalbertodt, submitted)^5^ tested whether naive readers would identify written RDs and ATs correctly. As a measure for correct identification, the prosodic realization of the read data was inspected and compared to the prosodic realization of spontaneously uttered RD and AT. It turned out that readers could identify written RDs and ATs correctly, marking RDs with a weaker ip-boundary and reduced pitch range, while ATs were produced with a stronger IP-boundary. Interestingly, contrary to Lambrecht (2001), ATs also were found to reduce pitch range in the course of the disambiguating phrase. Nevertheless, this reduction was not as strong as it was in RDs. Yet the test material in this particular study was simply taken from novels and there were two explanations for these different prosodic realizations: Prosodic realization may have been cued (i) by the discourse-pragmatic differences between RD and AT (i.e., referent continuity vs. ambiguity resolution respectively) or (ii) by differences in punctuation because RDs were consistently presented with a comma and ATs with a full stop. Both parameters are equally likely to affect prosodic marking, since they may be both connected to prosody, as explained above for discourse-structure.

As for punctuation, there are two different accounts on the nature of the German punctuation system that differ with regard to whether it is intonationally motivated (cf. e.g., Sappok, 2011) or syntactically motivated (cf. Primus, 2007). The first account proposes that intonation directly drives punctuation and, thus, the use of comma and full stop would mark intonational phrasing, especially the length of pauses between units. The full stop is associated with a longer pause, the comma with a shorter one. Since intonational punctuation approaches do not use the terms currently used in intonational research, we have to assume tentatively that these accounts would associate the full stop with the edge of an IP, and the comma with the edge of an ip. The second account, to the contrary, assumes that the punctuation marks under consideration are directly driven by syntactic structure. While the intonational account does not provide clear rules for punctuation, the syntactic account provides a set of three syntactic constraints that explain the whole comma system of German: the comma is licensed whenever (i) an expression is syntactically connected to the matrix structure and (ii) this connection is not a proper head-dependent relation. In German there is an additional constraint requiring (iii) a comma at the edge of an embedded clause. In the syntactic punctuation account, there is only an indirect link between punctuation and prosody, with both systems being affected by syntax (cf. Kirchhoff and Primus, 2014).

Regarding RDs and ATs, a previous, unpublished study by the first author showed that in novels, RD is separated by a comma from the matrix clause in 94.3% of the attested corpus [cf. example (1) above], while AT is set off by a sentence delimiting punctuation mark, i.e., a full stop or question mark, in 86.8% of the corpus [cf. example (2) above]. These findings are in accordance with current syntactic punctuation theories on German. Bredel (2011), for instance, points out that the full stop marks the end of a syntactic parsing unit, i.e., everything beyond the full stop does not belong to the previous syntactic structure. Because ATs are syntactically not connected to the matrix clause, as stated above, marking them with a full stop is appropriate. RDs, on the other hand, cannot co-occur with a full stop as they are integrated into the syntactic structure of the matrix clause. Instead, they may be separated from the matrix clause by a comma (Bredel, 2011). The reason is that the comma is licensed whenever an expression is syntactically connected to the matrix structure and this connection is not a proper head-dependent relation. However, this particular study was not designed to tease apart the effect of discourse structure and punctuation on prosody and to assess the exact link between prosody and punctuation.

In the current investigation, we seek to tease apart the contribution of punctuation and discourse on prosody by systematically crossing the two predictors. The aim of our current study is to investigate whether prosodic differences in RD and AT are triggered by punctuation or discourse structure. In order to solve this question we conducted a reading production experiment in which we combined two critical types of discourse structure (referential continuity vs. referential ambiguity) with two different orthographic realizations each (comma vs. full stop). There are three possible outcomes to this design: First, if punctuation has an immediate effect on the prosodic marking, full stops will lead to an IP-boundary, and mostly unreduced pitch register, while commas will result in an ip-boundary and reduced pitch range, irrespective of the underlying discourse structure. Second, if discourse structure leading to the syntactic choice of RDs vs. ATs has an immediate effect on the prosodic realization, then RD-contexts will lead to an ip-boundary with reduced pitch register, whereas AT-contexts will result in an IP-boundary without reduction of pitch range, irrespective of punctuation mark. Third, there could be an interaction of punctuation and discourse structure.

This experimental study will shed light onto the relation between punctuation, discourse (mediated by syntax) and prosody during the processing of RD and AT. It may also contribute to a better understanding of the architecture of writing systems in its relation to the architecture of language.

## Experiment

### Subjects

Twenty-four monolingual native-speakers of German (21 females) participated in this experiment after giving written informed consent. None of them reported hearing loss or speech disorder. The study was performed in accordance with the Declaration of Helsinki and with the national and institutional recommendations adopted by the Experimental Linguistics Lab in Cologne (XLinC). All subjects were paid for participation.

### Material and design

For our experiment, we created two lists with 8 ATs and 8 RDs each. In each list, four tokens of both RD and AT were assigned a comma and four tokens of each were assigned a full stop. Items that were assigned a comma in list one were presented with a full stop in list two and *vice versa*. The critical text items were adapted from corpus attestations in the *Harry Potter* novel series (Rowling, 1998-2007, translated by K. Fritz) and the German novel *Tintenherz* (Funke, 2003). They were controlled for length (number of words and sentences), complexity (mean number of clauses; cf. Kemper et al., 1989) and measures of coherence, i.e., number and type of referents (cf. Halliday and Hasan, 1976) to avoid systematic differences between the two constructions [see examples (7) for RD and (8) for AT]. Thus, RD items on average consist of 13.23 clauses and 69.875 words with an average of 10.875 occurring referents, while AT items on average consist of 13.86 clauses and 70.125 words with an average of 10.125 occurring referents.

1. RD
  Die **Niffler** tauchten in die Erde ein und wieder daraus auf, dann trippelte **jeder** zu seinem Schüler zurück und **ø** spuckte ihm Gold in die Hände. **Rons Niffler** hatte bald seinen ganzen Schoß mit Goldstücken gefüllt.
  “Kann man **die** auch als Haustiere kaufen, Hagrid?,” fragte Ron begeistert.
  “Das wär nicht so gut,” grinste Hagrid. “**Die** bringen ganze Häuser zum Einsturz, **diese Niffler**. Ich schätze, sie haben fast alle,” fügte er hinzu und ging um das Stück Erde herum. “Ich hab doch nur hundert Münzen vergraben.”
  “The **nifflers** disappeared into the earth and appeared again, then **each one** returned to his student and spat gold into his hands. Soon, **Ron's niffler** had filled his whole lap with pieces of gold.
  “Can you purchase **these** as pets, Hagrid?,” Ron asked excitedly.
  “That wouldn't be good,” smiled Hagrid. “**They** let whole buildings collapse, **these nifflers**. I guess, they almost got all [coins],” he added and walked around the piece of earth. “I buried only 100 coins.””
2. AT
  “Beim Namen Bode klingelt was bei mir…,” sagte Ron.
  “Wir haben ihn im St. Mungo gesehen, er lag nur da und hat an die Decke gestarrt,” flüsterte Hermine. “Und wir haben gesehen, wie *die Teufelsschlinge* ankam. ***Sie*** hat gesagt, es sei ein Weihnachtsgeschenk. **Die Heilerin**.”
  Harry sah sich den Artikel noch mal an und ein flaues Gefühl machte sich in seinem Magen breit.
  “Warum haben wir die Teufelsschlinge nicht erkannt?”
  ““The name Bode sounds familiar to me…,” said Ron.
  “We've seen him at St. Mungo's, he was just lying there, staring at the ceiling,” Hermione whispered. “And we've seen how *the Devil's Snare* arrived. ***She*** said it was a Christmas gift. **The healer**.”
  Harry read the article again and his stomach contracted.
  “Why didn't we recognize the Devil's Snare?””

Additionally, each list contained 16 filler items. The fillers serve as a baseline measure to assess the impact of comma and full stop on prosody because we are not aware of any studies that have tested the immediate prosodic consequences of punctuation at right clause boundaries. These fillers consist of 8 minimal pairs of coordinated root clauses, i.e., root phrases, as in (9) and (10), one version of each minimal pair marked with a full stop, the other marked with a comma:

1. Ron rüttelte ein wenig am Steuer, Harry schlug auf das Armaturenbrett.
2. Ron rüttelte ein wenig am Steuer. Harry schlug auf das Armaturenbrett.
  “Ron joggled the steering wheel. Harry hit the dashboard.”

According to recent punctuation theory (Primus, 2007; Bredel, 2011; Kirchhoff and Primus, 2014), the two clauses in (9) have to be interpreted as syntactically connected by a syntactic relation that is not a proper head-dependent relation. These features are also found in RDs. The two clauses in (10), on the other hand, have to be interpreted as syntactically disconnected. Syntactic disconnection also characterizes ATs. Considering Align-RootP at the prosody-syntax-interface mentioned above, we expect the fillers in (10) to be realized as two separate IPs. The prediction for the fillers with comma in (9) is less obvious because the two root clauses are connected, i.e., coordinated syntactically and the status of the resulting phrase is unclear. Truckenbrodt (2005) assumes that it is a coordination (boolean) phrase. Under this analysis, there will be no superordinate IP in (9) and no intonational difference to (10) since the relevant alignment constraints only hold for clauses, including root phrases, in German. However, some speakers may interpret (9) as one superordinate root phrase. In this event they are likely to produce only one IP for the superordinate root phrase degrading the underlying IPs for the deeper embedded main clauses to intermediate phrases (ip) or erasing them altogether. This degrading is explicable by the fact that stacking of intonation phrases is strongly dispreferred (cf. Truckenbrodt, 2007). Concerning pitch range and accent strength, we are not aware of any previous studies investigating such minimal pairs.

### Data acquisition and analysis

Each participant read aloud one of the two lists, containing 32 test items (16 critical and 16 fillers). Lists were presented on a computer screen, showing only one test item at a time. Subjects were instructed to read out the texts *immediately* when they appeared on the screen, in order to maintain the very first interpretation of the texts. In case of misreading, participants were told to repeat the whole sentence, in order to minimize the amount of non-intentional phrase breaks. To reduce mispronunciation, each participant received a list of unusual names (e.g., *Niffler*) right before the experiment, which they had to read aloud to familiarize themselves with these names. Subsequent to the production part of the experiment, subjects were asked to fill in two forms: in the first form they had to answer content questions on the test items. This questionnaire was given so participants would read the test items carefully enough to understand their discourse structure. No participant had to be excluded due to a high number of incorrect responses, as all of them answered at least 75% of the questions correctly. The second form included personal data and, additionally, ensured that none of the participants was aware of the experiment aim by asking what they thought had been investigated. Evaluation of questionnaires proved that none of our subjects was aware of the object of investigation.

Recordings were carried out with a headset in a soundproof booth, with a 44,100 Hz sampling rate and 16 bit resolution. Session length amounted to approximately 30 min per participant. For analyses, the data were coded for prosody by two independent GToBI-trained annotators who were unaware of the test condition, determining (i) accent strength and (ii) phrasing—following GToBI conventions (Grice and Baumann, 2002)—, and (iii) the pitch range of each token was calculated using Praat (Boersma and Weenink, 2015).

In annotating phrasing, transcribers could choose one of three labels: no boundary, (weaker) ip-boundary, and (stronger) IP-boundary immediately preceding the dislocated constituent. Also for accent strength, three labels were available: no prominence, postnuclear prominence, and nuclear prominence. Accent strength in this experiment was only determined for the dislocated phrase, as RDs and ATs are claimed to differ especially in this criterion (cf. Lambrecht, 2001). Thus, the assignment of pre-nuclear prominences was not possible as the dislocated phrase was always in final position. Cases of disagreement between the coders were resolved by discussion.

**Figure 3:**
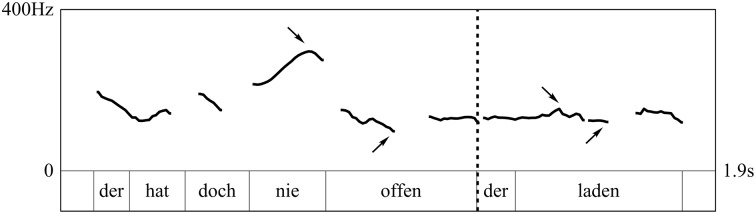
**Tonal contour of a RD test item (translation: “It's never open, this store”)**. The graph shows the f0-contour and the word level annotation. The dashed line marks the syntactic break, arrows indicate minima and maxima in pitch.

In order to avoid gender-dependent values in pitch range, the measure of this parameter was carried out as follows (see Figure 3). In a first step, the maximum and the minimum pitch of each syntactic phrase were extracted using Praat (Boersma and Weenink, 2015). In a second step, these extracted Hertz-values were transformed to semitones (st). Finally, we subtracted the pitch range-value of the matrix sentence from the pitch range-value of the dislocated phrase (in case of fillers, pitch range of RootP_1_ was subtracted from pitch range of RootP_2_) to calculate the pitch range-difference between the first and the second target phrase for each token. Thus, negative values represent a decrease in pitch range while positive values display an increase of pitch range toward the second (or dislocated) phrase.

### Statistical analysis

In total, 768 tokens (24 participants × 32 items) were analyzed: 384 filler tokens, 192 RD-tokens and 192 AT-tokens. For pitch range, a linear mixed effects model was run with the factors context type and punctuation mark (including possible interaction) as predictors and random intercepts for both speaker and item. For phrasing and accent strength in critical items, loglinear analyses were performed with the predictors context type and punctuation mark (including possible interaction). For phrasing in filler items, also a loglinear analysis was performed, where punctuation mark entered the model as predictor. Statistical analyses were done using the lme4-package (Bates et al., 2015) and the MASS-package (Venables and Ripley, 2002) in R (R Core Team, 2015).

### Results

Results will be presented for fillers and critical items separately. We will start with the presentation of the filler items, since they served as a baseline measure for the impact of punctuation on prosody.

#### Filler items: Main clauses

##### Pitch range reduction

**Figure 4:**
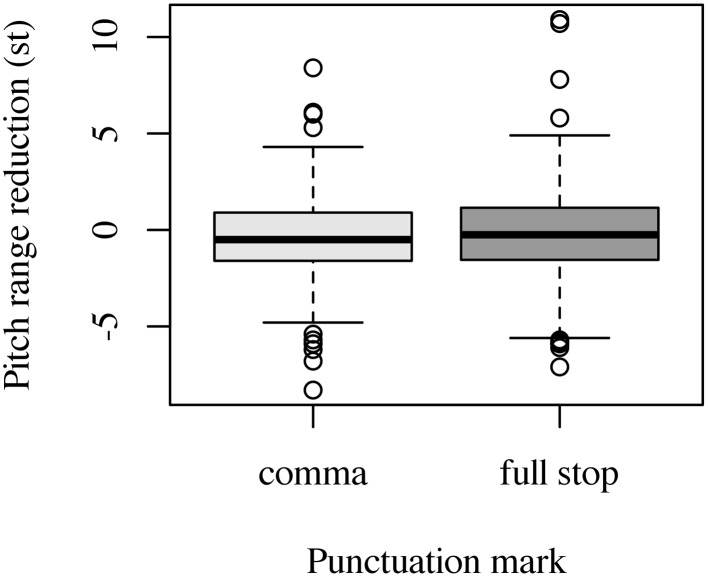
**Effect of punctuation mark on pitch range reduction (in st) in filler items**. Negative values indicate a decrease of pitch range, positive values an increase of pitch range.

In filler items, pitch range very slightly decreased from RootP_1_ to RootP_2_ in both comma condition (–0.37 st) and full stop condition (–0.2 st). In neither the comma condition nor the full stop condition was this decrease significant [comma condition: *t*_(381, 22)_ = 1.74, *p* = 0.08; full stop condition: *t*_(378, 78)_ = 0.87, *p* = 0.38]. As Figure 4 shows, there is nearly no difference between fillers marked with a comma and fillers marked with a full stop. This was supported by our linear mixed effects model where punctuation mark entered as factor and random intercepts were used for speaker and item. Our model did not reveal an effect of punctuation (χ^2^(1) = 0.522, *p* = 0.47).

##### Phrasing

**Figure 5:**
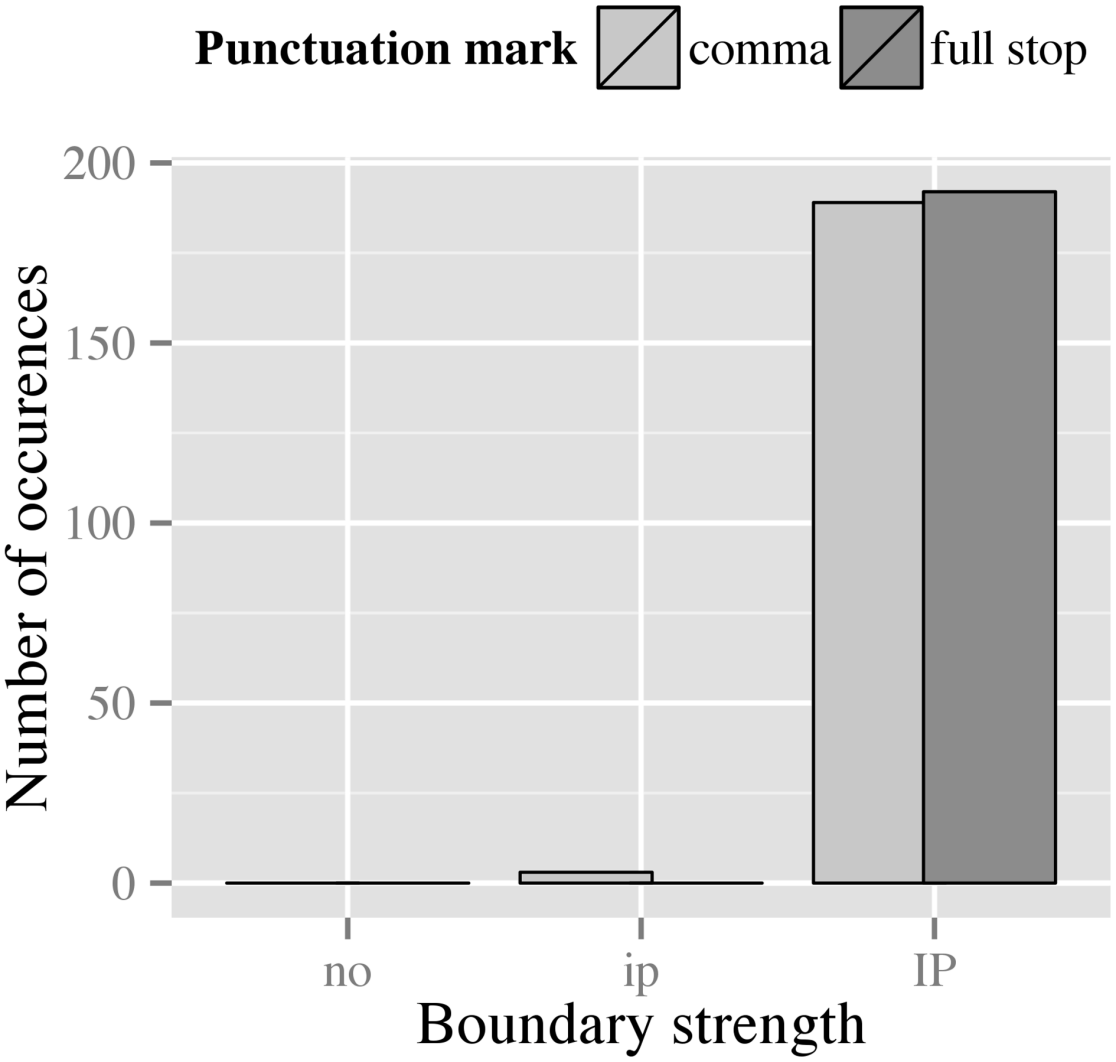
**Distribution of boundary types preceding the second phrase in filler items for both punctuation conditions**.

The clear majority of our data (99.2%) was produced with IP-boundaries between the coordinated clauses, while the rest (0.8%) was produced with an ip-boundary. As shown in Figure 5, realizations of filler items only slightly differed with respect to the distribution of boundary types. The figure suggests a trend for fillers marked with a full stop to be more likely to be produced with an IP-boundary than fillers marked with a comma. We consider this merely a trend because the loglinear analysis of the data is complicated by the fact that we do not observe any ip-boundary in the full stop condition, which leads to an infinite odds ratio. Employing the *ad-hoc* analysis suggested by Agresti (1996), often referred to as the delta option, did not reliably improve the interpretability of the odds ratio.

##### Accent strength

**Figure 6:**
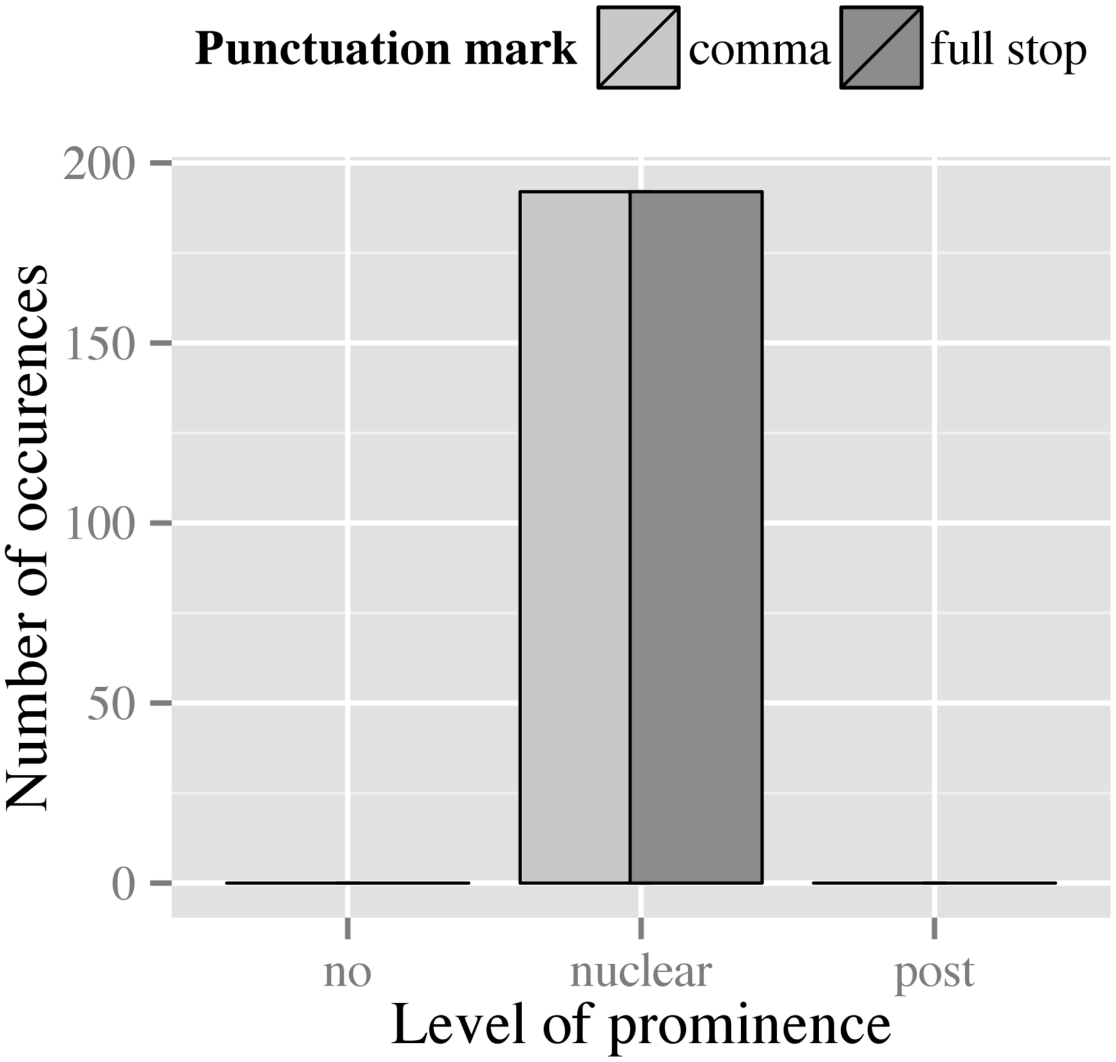
**Distribution of prominence levels assigned to the second phrase in filler items across both punctuation conditions**.

All of our data were produced with a nuclear prominence in the second RootP. This is due to the fact that in all items a phrase boundary preceded the second RootP. Hence, no effect of punctuation on prominence was observed, as displayed in Figure 6.

To sum up, in filler items merely the parameter of phrasing was marginally affected by punctuation with full stops receiving slightly more IP-boundaries than commas. Neither accentuation nor reduction of pitch range was affected by punctuation.

#### Critical items: RD vs. AT

##### Pitch range reduction

**Figure 7:**
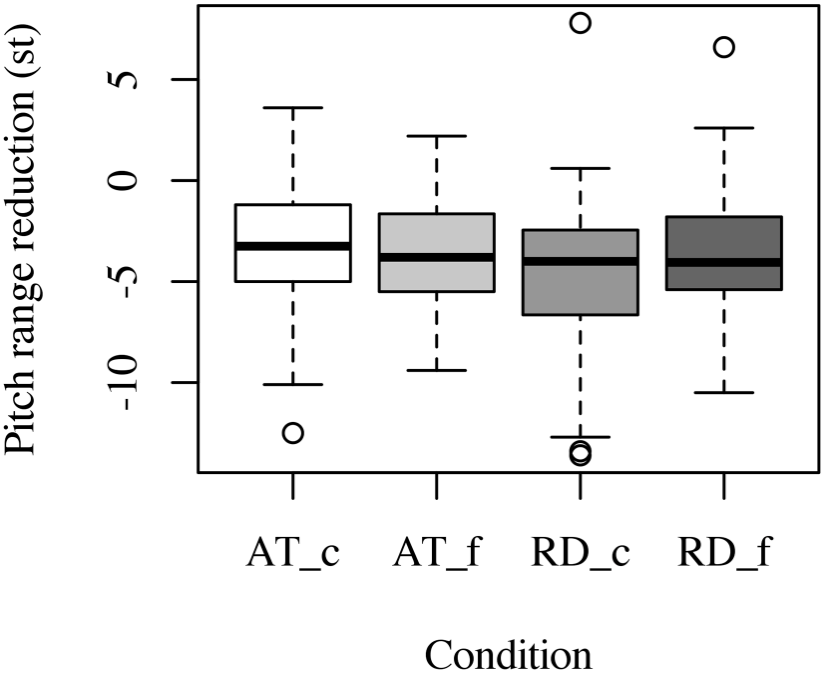
**Differences in pitch range reduction (in st) between RD and AT across punctuation conditions**. The letters c and f code the experimental condition, i.e., comma condition (_c) and full stop condition (_f).

In both RD and AT, participants used a reduced pitch range when producing the dislocated phrase of the target sentence. Although RDs are more reduced in pitch rage (-4.6 st in the comma condition, -3.9 st in the full stop condition; a decrease of 54.1 and 46.3%, respectively) than ATs (-3.4 st in the comma condition, -3.5 st in the full stop condition; a decrease of 41.7 and 42.1%, respectively; see Figure 7), this difference does not reach statistical significance. No effect of context type or punctuation mark could be confirmed. Context type did only have an impact on the reduction of pitch range when comparing critical items against fillers, where the former showed more reduction than the latter [**χ**^2^(2) = 35.86, *p* < 0.01].

##### Phrasing

**Figure 8:**
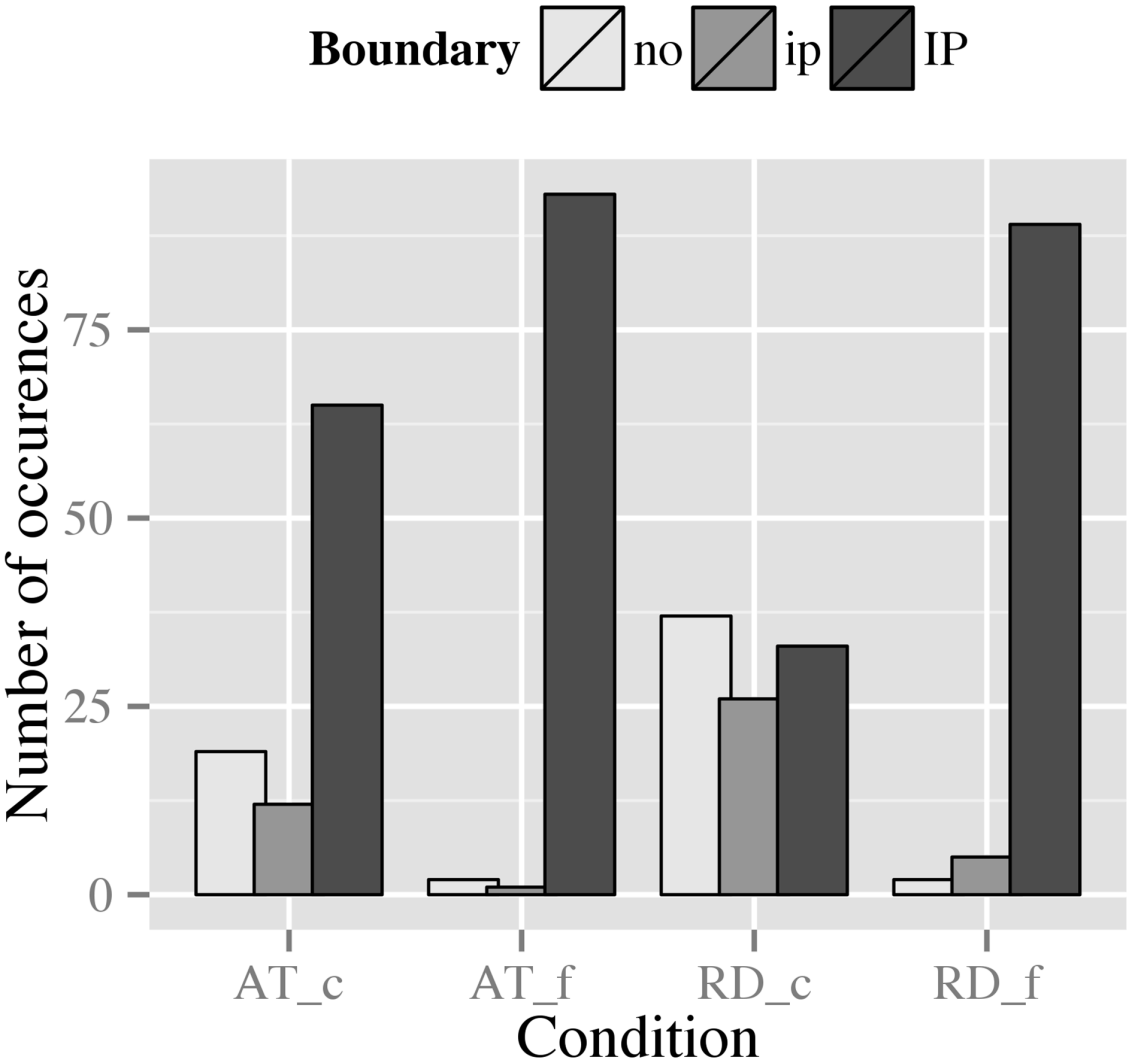
**Distribution of boundary types preceding the dislocated phrase in RD and AT for both punctuation conditions (_c, comma condition; _f, full stop condition)**.

If we compare RDs and ATs with respect to their distributions of boundary strength (Figure 8), two influences can be discovered: First, context type and the context-driven choice of the syntactic construction seems to affect the choice of boundary, as there are generally more IP-boundaries for AT than for RD; second, punctuation mark seems to have an effect on the variability of boundary strength within each construction, indicating an interaction of context type/syntax and punctuation mark. In the following we resolve this interaction by context.

RDs show a high degree of variability for boundary strength in the comma condition: 38.5% of the tokens were produced without a boundary preceding the dislocated NP, while 27.1% were produced with an ip-boundary preceding the NP, and 34.4% were produced with an IP-boundary. In the full stop condition, contrastingly, only 2.1% of the tokens were produced without a boundary and 5.2% were produced with an ip-boundary, whereas the remaining 92.7% of the tokens were produced with an IP-boundary preceding the dislocated NP (cf. Figure 8). Thus, the data display an effect for punctuation mark within RDs.

ATs overall show less variability for boundary strength (Figure 8). In the comma condition, 19.8% of the tokens were realized without a boundary preceding the clarification phrase, 12.5% were produced with an ip-boundary, and 67.7% were realized with an IP-boundary. Similar to RD, the observed variability decreases even more in the full stop condition, i.e., merely 2.1% of the tokens were realized without a phrase break, 1.0% was produced with an ip-boundary, and 96.9% were produced with an IP-boundary. These patterns indicate an effect of punctuation mark on the choice of boundary strength within ATs.

For the statistical analysis of this categorical data output, we used loglinear analysis, which is a generalized form of a chi-square test. As in chi-square testing, the observed values are compared to so-called expected values^6^. If the observed values differ significantly from the expected ones, this indicates that observed values are not due to chance but rather that there is an effect of the test variable. To compute the effect size of the test variable, a model is produced in a first step that retains all predictors and thus fits the data perfectly (the saturated model). Next, the model is reduced stepwise by removing interactions of predictors, and then by removing main effects. Pairwise comparisons of the saturated model and the reduced models reveal which model fits the data best. Important for this best-fit judgment is the *p*-value: note that, opposed to other statistical tests, in loglinear analysis a *p* > 0.05 is desirable as it tells us that the model is not significantly deviating from the observed data. Thus, the last non-significant model produced by loglinear modeling is used for data analysis (cf. Field et al., 2012). For boundary strength our loglinear analysis produced a final model with a likelihood ratio of **χ**^2^(2) = 1.48, *p* = 0.48, which retained all effects. This model confirms our tentative findings that there is an interaction of context type and punctuation mark [**χ**^2^(1) = 5.44, *p* = 0.02] affecting boundary strength. Additionally, we observe main effects for context type [**χ**^2^(2) = 21.91, *p* < 0.01] and punctuation [**χ**^2^(2) = 112.58, *p* < 0.01].

##### Accent strength

**Figure 9:**
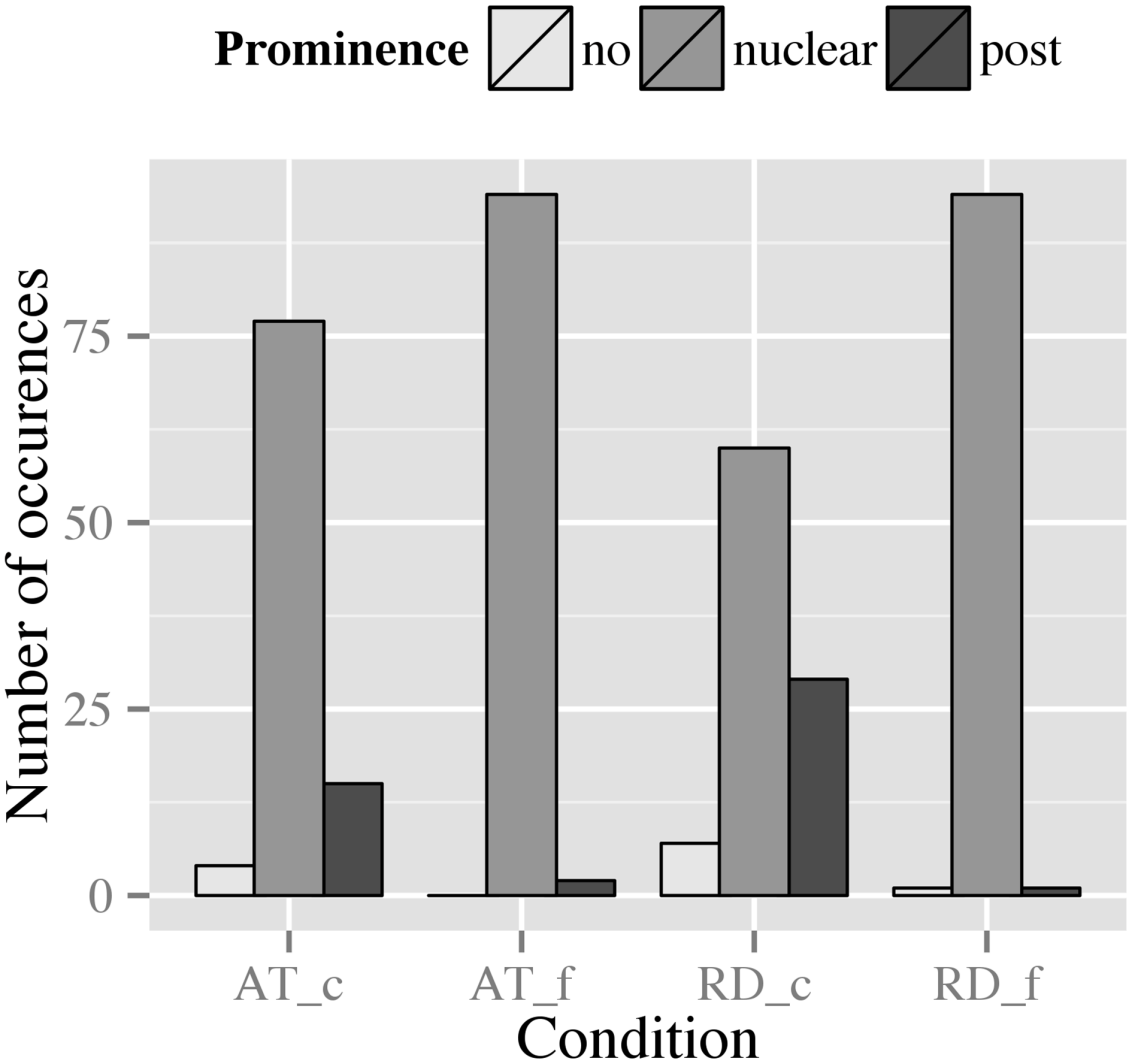
**Distribution of prominence levels assigned to the dislocated phrase in RDs and ATs across punctuation conditions (_c, comma condition; _f, full stop condition)**.

When comparing RD and AT with respect to the prominence levels involved, Figure 9 suggests slight effects of context type and punctuation mark, since RDs in the comma condition pattern differently from the other conditions.

For RD, a similar pattern as for boundary strength can be observed (Figure 9): in the comma condition we find a high degree of variability with 7.3% of the tokens being deaccented, 30.2% being realized with a postnuclear prominence and 62.5% being realized with a nuclear accent on the dislocated NP. In the full stop condition, however, this variability decreases: merely 1.0% of the tokens was deaccented, 1.0% was realized with a postnuclear prominence, and 97.9% realized with a nuclear accent. These patterns suggest an effect of punctuation, i.e., that the full stop leads to a preference of a strong(er) prominence regarding the accentual marking of the dislocated NP.

For the prominence levels produced in ATs (Figure 9), we observe a similar pattern as for boundary strength: in the comma condition, 4.2% of the tokens were deaccented, 15.6% were realized with a postnuclear prominence, and 80.2% were realized with a nuclear accent. In the full stop condition, to the contrary, merely 2.1% of the tokens were realized with a postnuclear prominence, while the remaining 97.9% of the data were produced with a nuclear accent. Again, this pattern seems to indicate an effect of punctuation, i.e., that marking of the dislocated constituent by a nuclear accent is strongly favored in the presence of a full stop.

Our loglinear analysis produced a final model with a likelihood ratio of **χ**^2^(3) = 3.31, *p* = 0.35, which only contained main effects for context type [**χ**^2^(2) = 6.81, *p* = 0.03] and punctuation mark [**χ**^2^(2) = 61.54, *p* < 0.001].

To sum up, pitch range reduction in RD and AT did not differ significantly and thus was neither affected by context type nor by punctuation mark. Phrasing, however, was influenced by an interaction of context type and punctuation mark. Accent strength was affected by both context type and punctuation mark.

## Discussion

### Main clauses

Previous accounts have formulated both direct (e.g., Sappok, 2011) and indirect correspondences (e.g., Primus, 2007; Kirchhoff and Primus, 2014) between punctuation and prosody but no empirical evidence has yet been presented. Filler items in this experiment were used as a baseline measure to assess the immediate effect of punctuation on prosody with clausal constituents. We constructed sentences consisting of two main clauses and created minimal pairs by simply changing the punctuation mark between them from comma to full stop. As there are two diverging views on punctuation, one intonationally motivated, the other syntactically motivated, investigation of the prosodic realization of these minimal pairs sheds light on the nature of the link between prosody and punctuation. As mentioned in the Introduction, the intonational punctuation account assumes a direct link between prosody and punctuation in that punctuation marks are claimed to code pauses of differing length between units (cf. e.g., Sappok, 2011). Following this assumption, a comma would lead to a minor break (ip) between the two main clauses, whereas a full stop would result in a major break (IP) between them and a (longer) pause. Thus, there would be an immediate effect of punctuation. The syntactically motivated punctuation theory, in turn, proposes an indirect link between prosody and punctuation: Both systems receive the same input from syntax, resulting in a mere correlation of punctuation and prosody (cf. Primus, 2007; Kirchhoff and Primus, 2014).

Our results for filler items revealed only a very small difference in the realization of main clauses separated by a comma as opposed to main clauses separated by a full stop. As such, pitch range and accent strength have not been affected by punctuation mark. However, the choice of prosodic boundary has been slightly influenced by punctuation: fewer tokens were produced with an IP-boundary in the comma condition. Our findings for the full stop can be explained both syntactically and prosodically since there is a particularly tight link between syntax and prosody in this case. The full stop forces an interpretation of the two main clauses as syntactically disconnected, as mentioned in the results section for filler items above; prosodically, two separate root clauses form an IP each (see Align-RootP). Our results in the comma condition can be explained syntactically as follows: Truckenbrodt (2005) assumes that coordinated main clauses form a coordination (boolean) phrase. Under this analysis, there is no intonational difference from the full stop condition since the relevant prosody-syntax alignment constraints only hold for embedded or root clauses in German. However, some participants may have interpreted syntactically connected main clauses as one superordinate root phrase. In this event they are likely to produce only one IP for the superordinate root phrase degrading the underlying/potential IP for the embedded clause to ip or erasing it altogether.

In conclusion, the weaker or stronger prosodic boundaries triggered by the punctuation marks under discussion seem to be a consequence of the absence or presence of a syntactic connection between main clauses. The full stop prohibits a connection and always correlates with a strong boundary (IP). The comma, by contrast, requires a syntactic connection. Depending on the way speakers interpret syntactically connected main clauses, the comma will trigger a stronger boundary (IP), a weaker one (ip) or no boundary. By contrast, the intonational comma approach makes the wrong prediction that the comma is invariably associated with a weaker prosodic boundary.

### RDs and ATs

We first summarize the findings for the different conditions investigated in this study. For RDs in the comma condition it could be observed that pitch range was significantly reduced in the dislocated phrase, while the patterning regarding boundary strength was quite variable. Nevertheless, the majority (65.6%) of tokens was produced as a single IP. Also in terms of accentuation, a high degree of variability could be observed. However, the majority of tokens was marked by a nuclear accent. RDs in the full stop condition showed a significant amount of pitch range reduction in the dislocated phrase, while the pattern for boundary strength clearly favored the presence of an IP-boundary (92.7%), resulting in two separate IPs. A similar result was observed with respect to accentuation, where the data were also less variable and favored marking of the dislocated phrase by a nuclear accent. Regarding ATs in the comma condition, a significant reduction of pitch range in the dislocated phrase was observed, while two thirds (67.7%) of the data were set off from the matrix sentence by an IP-boundary, resulting in two separate IPs. The majority of the tokens was realized with a nuclear accent on the dislocated phrase. Also in the full stop condition, ATs were found to significantly reduce pitch range and strongly favor an IP-boundary (96.9%) and prosodic marking by a nuclear accent. The most striking difference between the comma condition and the full stop condition was the lack of variability in the latter.

Overall, both RD and AT significantly reduced the pitch range in the dislocated constituent compared to fillers. However, the amount of reduction in RD and AT did not significantly differ, thus, no effect of context type (mediated by syntax) or punctuation mark could be observed within critical items. This result is particularly interesting as it contrasts with Lambrecht's (2001) claim that pitch range is only reduced for RD but not for AT. It also counters our expectations based on discourse context, where we anticipated an increase of pitch range for ATs rather than a decrease. However, the results presented here coincide with the results of a previous investigation: a corpus study by Kalbertodt and Baumann (2015) found pitch range reduction in spontaneously uttered RDs and ATs; however, the difference in the amount of reduction (more reduction in ATs) was not statistically reliable either. These findings need to be addressed by future research to explore the role of pitch range reduction in the distinction of RD and AT. Since a difference in pitch range is treated as a key difference between RD and AT in the literature (cf. Lambrecht, 2001; Dewald, 2014), we suggest a perception study regarding this parameter. This perception study could investigate whether or not reduction of pitch range serves as a necessary cue for listeners to recognize RD and AT correctly, and to which extent pitch range has to be reduced in RD and AT for correct recognition.

Concerning phrasing, our statistic model confirmed an interaction effect of context type/syntax and punctuation mark. Recall that we expect ATs to be realized as two separate IPs whereas RDs should be realized as a single IP. If we now look at our data again, it becomes evident that these predicted patterns only hold for ATs and RDs in the comma condition. In the full stop condition, however, RDs and ATs show a similar pattern, favoring the production of IP-boundaries. This indicates that punctuation is a strong predictor for phrasing.

These patterns can be explained by the interface constraints presented in the Introduction and by assumptions about cue cost and cue reliability (cf. Bates and MacWhinney, 1989) as follows. Let us start with the full stop condition in which RD and AT show a similar 2-IP pattern. As discussed above, the full stop unequivocally signals the end of a root phrase. Its prosodic correlate is therefore invariantly an IP-boundary (as predicted by Align-RootP). It is therefore a very reliable cue. It is also a local cue since its value (or function) can be immediately assigned when it is encountered. Since the discourse structural cues for RD vs. AT are very subtle and global, i.e., are computationally more demanding and have to be stored in working-memory, it is plausible to assume that the vast majority of participants was guided by the reliable and local full stop and neglected the more global contextual difference between RDs and ATs.

Let us now turn to the comma condition. The comma signals a syntactic connection leaving the status of this connection open. The comma is licensed by coordination and dislocation since they do not involve a head-dependent relation and, additionally, by an embedded clause boundary in German (cf. Primus, 2007; Kirchhoff and Primus, 2014). Coordinations and root phrase dislocations determine IP-boundaries and this also holds for the right edge of embedded clauses in German (cf. Align-Clause in Truckenbrodt, 2005; Selkirk, 2011). Therefore, it is not surprising that roughly one third of all RDs separated by a comma were produced with an IP-boundary before them, as prompted by the potential prosodic correlate of this local punctuation cue. In order to produce a RD with an ip-boundary or no prosodic boundary, participants had to consider the preceding context and the whole construction, i.e., global cues, particularly the fact that matrix clause and RD form a syntactic unit that licenses one IP, as explained in greater detail in the Introduction above. Roughly two thirds of all RDs were produced by honoring the global cue and by interpreting the local cue in accordance with the global cue. Recall that the comma is also compatible with no boundary or a weaker boundary. With ATs we find a mirror image. Roughly two thirds of all ATs were produced in accordance with the global cue, i.e., as two IPs. The local cue has been interpreted in accordance with the global cue since the comma is compatible with this intonation pattern. Only roughly one third of the ATs were produced by only taking heed of the local cue, which is compatible with the absence of a 2-IP prosodic pattern. In sum, the variation in the comma condition can be explained by our constraints and by assumptions about cue cost, i.e., that local cues may override global ones when there is a competition between them because they are less costly.

Finally, let us explain the difference between the two local cues, the full stop and the comma, when there is a competition between the local and the global cue. This occurs when contextually prompted RDs are separated by a full stop and when contextually determined ATs are set off by a comma. A competition also seems to be at stake when RDs separated by a comma exhibit a 2-IP pattern because this pattern is compatible with the comma but incompatible with the contextually driven interpretation. In competition with the global cue, the full stop seems to have a stronger effect: 92.7% of the RDs exhibit a 2-IP pattern prompted by the local full stop. But only 32.3% of the ATs exhibit the lack of an IP-pattern (an ip or no boundary) as prompted by the local comma. Similarly, 34.4% of the RDs in the comma condition exhibit a 2-IP pattern that is only compatible with the local comma. The difference between the two local cues is explicable by cue reliability: prosodically unequivocal cues such as the full stop are more reliable and may show a stronger effect in competition with global cues than prosodically vague cues such as the comma.

The results for accent strength show an effect of both context type and punctuation mark. RDs in the comma condition show a high degree of variability, in contrast to ATs in the comma condition. For both constructions, however, the majority of tokens was produced with a nuclear accent on the dislocated phrase. In the full stop condition, this variability is no longer existent. Although an effect for punctuation mark has been found it has to be mentioned that the appearance of a nuclear accent is strongly connected to the presence of an ip- or IP-boundary before the dislocated constituent. As Crystal (1969) pointed out, an ip has to contain one nuclear accent and thus the nuclear accent is automatically assigned to the dislocated phrase if it was preceded by an ip- or IP-boundary. Following this, it could be argued that we did not observe an *immediate* effect of punctuation mark on accentuation but rather an indirect effect, since punctuation mark influenced the choice of boundary. Therefore, the explanation we offered for the selection of boundary type may be carried over to accent choice.

Overall, the greater impact of punctuation marks on prosody to the detriment of discourse structure is best explained by the assumption that local cues (punctuation marks) may override global ones (context) if they compete with each other. This seems to have happened in roughly one third of the critical items in our experiment in the comma condition and in the vast majority of cases in the full stop condition. A direct connection between the punctuation marks under discussion and prosody is not needed. Moreover, an intonational account of the comma predicts an invariant prosodic realization that is incompatible with our findings. By contrast, a syntactic account of full stop and comma in conjunction with assumptions about their processing cost and reliability captures our data more appropriately.

Our type of explanation seems, *prima facie*, to be incompatible with the neurophysiological study of Steinhauer and Friederici (2001). They show that the recognition of a comma in silent reading correlates with a Closure Positive Shift (CPS). This is an event-related potential (ERP) effect that is also found at prosodic boundaries in spoken language. The authors conclude that their findings suggest a direct link between comma and prosody. However, as argued by Kerkhofs et al. (2008), this assumption is not needed. Their ERP-data are best explained by assuming—as we do—that comma and prosodic boundaries have similar functions as markers of syntactic boundaries. The developmental ERP-study of Männel and Friederici (2011) supports this assumption. They show a strong interaction between the development of syntax and the CPS-effect. Only children with a highly developed syntactic competence exhibit a prosodic CPS-effect in their study. Eye-tracking during silent reading of syntactically ambiguous phrases in English also corroborates a direct link between syntax and comma (Hill and Murray, 2000).

## Conclusion

In this reading experiment we manipulated the constructions RD and AT with respect to the (mis-)match between discourse structure and punctuation in order to investigate the impact of these parameters on prosodic realization. As correlates of prosody we measured reduction of pitch range, boundary strength (phrasing) and level of prominence (accentuation). In addition, filler items consisting of two main clauses served as baseline measure to assess the impact of punctuation on prosody in the absence of discourse manipulation.

Our results for fillers revealed only a very small difference in the phrasing of main clauses separated by a comma as opposed to main clauses separated by a full stop: fewer tokens were produced with an IP-boundary in the comma condition. However, the assessment of the results' significance appeared rather problematic, and therefore it remains controversial whether the observed effect is sufficient to draw further conclusions. Extending the measures taken here by the parameters of pause duration and boundary tone, where systematic changes due to punctuation are expectable, might provide further evidence for the presence or absence of an effect driven by punctuation.

Concerning our critical data, the measure of pitch range did not reveal any effect of discourse or punctuation. This adds to the mixed findings in previous studies. Future research needs to address the question which role the reduction of pitch range plays in the marking of RD and AT, especially as the pitch range was reduced for critical items but not for filler items. A perception experiment, for example, could explore to which extent listeners rely on this prosodic cue to distinguish RD from AT.

Regarding prosodic phrasing and accentuation, results of our critical data revealed an impact of both discourse structure and punctuation. The results suggest that prosodic phrasing is directly determined by syntactic phrasing and by discourse, i.e., information structural differences between RD (comment-topic structuring) and AT (referential ambiguity resolution). The latter determines (de-)accentuation, which in turn may override syntactic alignment constraints on phrasing. Both comma and full stop are sensitive to syntactic phrasing and this explains their indirect impact on prosody and their interaction with discourse structure, i.e., the contextually driven choice of RD or AT. Our explanation for the prosodic patterns of critical items also includes general processing assumptions about cue cost and cue validity in order to explain the interaction between punctuation marks and context information. Thus, this explanation supports the syntactic account of full stop and comma and challenges intonational accounts (e.g., Sappok, 2011). The prosodic variation in the comma condition we found in the critical items (and fillers), in particular, is an important evidence for our account and against an intonational approach. On a more general methodological level our study shows that punctuation is a factor that cannot be neglected in experimental linguistics.

Regarding language architecture, our overall results are best explained by assuming a model in which punctuation, prosody, syntax, and discourse-semantics are independent but interacting domains with correspondence constraints between them. Our further assumption is that certain correspondence constraints are particularly strong. On the basis of the current data these are the correspondences between (i) punctuation and syntax, (ii) prosody and syntax as well as (iii) discourse-semantics and syntax. This means that syntax acts as a mediator between the other domains. Prosody, (de-)accentuation, i.e., prosodic prominence, in particular, is also determined by information structure. These assumptions are in line with many, but not all current approaches to the domains under consideration. Specifically, our results challenge approaches that posit a direct link between prosody and punctuation. Our study contributes to a better understanding of the architectural design described above by including punctuation and by fuelling the discussion about the interaction of the domains under consideration with new experimental data.

## Author contributions

JK designed the experimental task, conducted the experiment, ran the statistical analysis and wrote the article. PBS designed the experimental task and wrote the article. BP designed the experimental task and wrote the article.

### Conflict of interest statement

The authors declare that the research was conducted in the absence of any commercial or financial relationships that could be construed as a potential conflict of interest.

## Footnotes

^1^In our examples of German RD and AT, the target referent is marked with bold print and the target sentence is underlined. Competing referents are italicized [in (5)]. Also note that examples (4) and (5) are taken from the database Fokus-DB (http://www.linguistik.net/cgi-bin/focus.pl) that contains occurrences of (semi-)spontaneously uttered peripheral constructions. Thus, these examples are non-punctuated transcripts of real speech, where the dots indicate pause length.

^2^For a more detailed description of phrase structure and boundary strength see Shattuck-Hufnagel and Turk (1996).

^3^This example is taken from the Fokus-db (http://www.linguistik.net/cgi-bin/focus.pl).

^4^This example is taken from the Fokus-db (http://www.linguistik.net/cgi-bin/focus.pl).

^5^Kalbertodt, J. (submitted): Right dislocation and afterthought in novels: an empirical study on German. *Linguistische Berichte*.

^6^Expected values are calculated on the basis of the observed values, by multiplying the row sum and the column sum and dividing this result by the total number of observations.
